# Correction to “Methotrexate‐Induced Drug Hypersensitivity Syndrome Presenting With Acute Cerebral Infarction in End‐Stage Renal Disease: A Case Report”

**DOI:** 10.1002/ccr3.73427

**Published:** 2026-08-30

L. Mu , C. Yu , J.‐Y. Shi , et al., “Methotrexate‐Induced Drug Hypersensitivity Syndrome Presenting With Acute Cerebral Infarction in End‐Stage Renal Disease: A Case Report,” Clinical Case Reports 14, no. 7 (2026): e73142, 10.1002/ccr3.73142.42445457 PMC13357680

Affiliation 1 was incorrect and has been corrected to “Hangzhou Hospital of Traditional Chinese Medicine, Zhejiang Chinese Medical University.” In addition, Affiliation 2 has been removed from the affiliations of Le Mu and Cao Yu.

The online version of this article has been corrected.

We apologize for this error.

